# Featured Abstracts From the 19th National Conference on Chronic Disease Prevention and Control

**Published:** 2005-03-15

If necessity is the mother of invention, creativity in public health has never been more important. Fortunately, the ability of the public health *thinkforce* (as compared to workforce) to respond to the challenges inherent in assuring the public’s health is remarkable. The willingness of the thinkers to share information has always been a strength of the field, and now new technologies have enhanced our abilities to communicate what works, for whom, and under what conditions.

Challenges to population health continue to mount. Risk factor increases (e.g., obesity) and poorer access to services (e.g., percentage of population without insurance) conspire with multiple other health determinants to create monumental challenges for public health, particularly in the area of health disparities. Understanding disparities — their roots and their implications — is a difficult challenge; our future success will be largely determined by our response to this challenge. Correcting disparities will require, in part, the best application of chronic disease program knowledge to the populations at greatest risk.

The planners of the 19th National Conference on Chronic Disease Prevention and Control invited state and federal public health leadership, academic researchers, and others to think about solutions for the disparities that exist and continue despite our efforts. Some of the most impressive responses to that invitation appear here. Creativity and curiosity are features of the work presented in this section, and, it is hoped, they will spark those virtues in the readership.

One year ago, shortly after the launch of *Preventing Chronic Disease*, the decision was made to incorporate the best abstracts from the annual Chronic Disease Conference as a regular feature. The abstracts capture the field of public health now, offering a glimpse of what is being done, what works, and how it does so. They come from state and local chronic disease prevention programs and the academic community, including the Prevention Research Centers. Furthermore, they reflect the seven conference tracks: Partnerships; Evidence-based Programs: Research, Translation, and Evaluation; Health System Change; Social Determinants of Health Inequities; Communications and Technology; Methods and Surveillance; and Policy and Legal. These abstracts represent some of the best current work in the field of public health.

Two years ago, those not participating in the conference would be hard pressed, by both time and access, to find this information. Today, this issue of *Preventing Chronic Disease* brings the information to your desktop. The abstracts can inform you, challenge you, and connect you to colleagues who share your interests. You are invited to take advantage of this opportunity and to react with your own ideas — those that best meet the needs of your communities.

