# Supplementary material for: Featured Abstracts From the 19th National Conference on Chronic Disease Prevention and Control
Source: Prev Chronic Dis. 2005 Mar 15;2(2):A21. (PMC1327715)
Supplement: Related texts

# Sub article: PE2GO: A Program to Address Disparities in Youth Physical Activity Opportunities

**Published:** 2005-03-15

**Authors:** Maurice W Martin, Sarah Martin, Elmer Ray Elmer

**Affiliations:** Centers for Disease Control and Prevention, National Center for Chronic Disease Prevention and Health Promotion, Division of Diabetes Translation

## Track: Partnerships

The purpose of this study was to investigate the effectiveness of the PE2GO pilot program in six school districts across the United States (Chicago, Ill; Los Angeles, Calif; Akron, Ohio; New York, NY; Memphis, Tenn; and Portland, Ore). PE2GO is a community affairs initiative of Nike, Inc, the athletic apparel and shoe manufacturer based in Beaverton, Ore. Within the PE2GO program, Nike partners with organizations across the country to offer programs in underserved areas (e.g., Native American Boys & Girls Clubs, African American and Latino communities in Los Angeles) to foster physical activity among youth through their influencers such as parents, teachers, and coaches. PE2GO is a self-contained physical education (PE) program that provides classroom teachers with the tools they need to lead developmentally appropriate, quality PE lessons in their fourth- and fifth-grade classrooms in inner-city schools. The pilot program reached 6000 elementary school students.

In September 2003, experienced trainers from nonprofit Sports, Play, and Active Recreation for Kids (SPARK) conducted a one-day training of PE staff using a playbook created especially for the PE2GO program. Nike provided the curriculum and the necessary equipment. The initial training focused on two themes: building a foundation and disguising fitness. A second training approximately four months later focused on a third theme: simplifying sports. Trained evaluation consultants independent from Nike or SPARK collected data for the program’s evaluation and analyses in three distinct phases: pre-intervention, mid-intervention, and post-intervention. The intervention occurred through May 2004, and all data were reported by the faculty and administrators at the schools where the curricula were implemented (N = 164); this group included classroom teachers (n = 128), PE specialists (n = 22), and school-level administrators (n = 14).

Reported minutes of PE per week increased significantly from pre-intervention to mid-intervention (37 minutes pre-intervention vs 60 minutes mid-intervention; *P* < .05) and remained significantly higher than pre-intervention at the post-test (73 minutes). Satisfaction increased significantly from pre-intervention to mid-intervention (*P* < .05) and remained elevated post-intervention. Four of the eight questions assessing barriers showed that barriers decreased significantly from pre-intervention to mid-intervention (*P* < .05) and remained reduced post-intervention. Almost all administrators reported that they would support staff development (94%) and encourage staff to implement PE2GO (88%); more than half said they would reward staff for implementing PE2GO with fidelity (56%). From the qualitative research, almost all responded that administrators have expressed support for the program, yet about half added that administrators have had little involvement. Classroom teachers were successfully trained and satisfied with the program and the effect it was having on their fourth- and fifth-grade students. Reported minutes of PE increased substantially.

The PE2GO program holds promise in this day of declining opportunities for children to be active during their school hours, especially in schools with limited resources for PE specialists.

# Sub article: Awareness of Cardiovascular Disease Risk in American Indians

**Published:** 2005-03-15

**Authors:** Carrie S Oser, Lynda Blades, Carol Strasheim, Steven Helgerson, Dorothy Gohdes, Todd Harwell

**Affiliations:** Montana Department of Public Health and Human Services

## Track: Methods and Surveillance

Although cardiovascular disease (CVD) has become the leading cause of death in American Indians, little is known about how Indian communities perceive their risk.

In 2003, a telephone survey was conducted among adult American Indians living on or near Montana’s seven Indian reservations. Respondents were asked about awareness of heart disease risk; history of cardiovascular disease (CVD) such as heart attack, angina, or stroke; and risk factors for CVD.

The prevalence of CVD and risk factors among men and women aged 45 years and older (N = 516) was high: CVD (26% in men and 15% in women), diabetes (24% in men and 26% in women), high blood pressure (48% in men and 46% in women), high cholesterol (34% in men and 40% in women), smoking (28% in men and 33% in women), and obesity (37% in men and 46% in women). Men with a history of certain medical conditions were more likely to be aware of their risk for heart disease than men without these conditions: CVD (87% with vs 46% without), high blood pressure (70% with vs 44% without), high cholesterol (71% with vs 53% without), and obesity (67% with vs 52% without). The same was true of women: CVD (98% with vs 58% without), diabetes (74% with vs 60% without), high blood pressure (73% with vs 56% without), high cholesterol (72% with vs 60% without), and obesity (74% with vs 55% without). Neither men nor women associated smoking with their own risk for heart disease.

The prevalence of CVD risk factors was alarmingly high in this population. Awareness of risk for heart disease was associated with most modifiable CVD risk factors, except smoking.

# Sub article: Race, Ethnicity, and Linguistic Isolation as Determinants of Participation in Public Health Surveillance Surveys

**Published:** 2005-03-15

**Authors:** Michael W Link, Ali Mokdad, Herbert Stackhouse, Nicole Flowers

**Affiliations:** Centers for Disease Control and Prevention, National Center for Chronic Disease Prevention and Health Promotion, Division of Adult and Community Health

## Track: Methods and Surveillance

Public health officials and researchers require valid and reliable public health surveillance data to plan, implement, and evaluate programs designed to eliminate health disparities among racial and ethnic minority populations. Monitoring chronic disease and behavioral risk factors among such populations, however, has proven challenging. This research is designed to assess disparities among minority populations in participation levels in public health surveillance efforts and to test alternative methods for reducing these disparities.

We analyzed data from the 2003 Behavioral Risk Factor Surveillance System (BRFSS), which is a monthly, random-digit–dialed telephone survey of the noninstutionalized adult (aged 18 years and older) population in the United States. County-level data from the 2003 BRFSS and 2000 U.S. Census are modeled using ordinary least squares regression to examine the effects of race, ethnicity, and linguistic isolation on six measures of survey participation (e.g., resolution, screening, cooperation, refusal, refusal conversion, response rates).

The study finds that even after adjusting for other factors such as socioeconomic conditions, average commute time, use of call screening technology, and level of data collection effort (other factors thought to be related to survey response), areas with higher percentages of African Americans (regression coefficient, −0.14, *P* < .001), Hispanics (regression coefficient, −0.57, *P* < .001), and those who do not speak English — particularly those speaking only Asian (regression coefficient, −1.67, *P* < .001) or Indo-European (regression coefficient, −2.73, *P* < .001) languages — were significantly less likely than whites to participate in the public health surveillance.

In response to this finding, the BRFSS is investigating two alternatives for reaching these underrepresented groups: 1) use of alternative survey modes; in particular, providing translated hard-copy versions of the BRFSS by mail, and 2) use of specialized language line translation services to offer real-time translation of the BRFSS into languages beyond English and Spanish.

The collection of valid and reliable data for public health surveillance in the United States is becoming challenging. Current methods increasingly underrepresent racial, ethnic, and linguistically isolated groups. As a result, the health problems and needs of these groups may be significantly underreported. The development of successful public health interventions and programs capable of reducing health disparities requires that monitoring systems be developed that are capable of tracking the public health of all groups.

# Sub article: Measuring Population Health Disparities: The *Wisconsin County Health Rankings*

**Published:** 2005-03-15

**Authors:** Angela M Kempf, Patrick L Remington, Paul E Peppard, Elizabeth A Dranger, David A Kindig

**Affiliations:** University of Wisconsin Medical School, Department of Population Health Sciences, Wisconsin Public Health and Health Policy Institute

## Track: Methods and Surveillance

The purpose of this project was to rank the population health of counties in Wisconsin to promote use of local population health information, identify disparities between counties, encourage population health improvement, and broaden the understanding of the determinants of health.

The Wisconsin Public Health and Health Policy Institute, with assistance from state government, community, and university groups, annually compiles county data and produces the *Wisconsin County Health Rankings* report.

This project is modeled after the United Health Foundation's annual *America's Health: State Health Rankings* and is based on a population health model in which a variety of determinants impact health outcomes. Mortality years of potential life lost (YPLL) and self-reported health status are used to develop a summary measure of county health outcomes. A summary measure of health determinants is developed using 18 measures in four (weighted) categories: health care (10%), health behaviors (40%), socioeconomic factors (40%), and physical environment (10%). Data sources include the Centers for Disease Control and Prevention, the U.S. Census, state vital statistics, and the Wisconsin Department of Health and Family Services. A draft report was developed and shared with local public health officials in late 2003. The report was revised on the basis of feedback, and *Wisconsin County Health Rankings, 2003* was released to the public in January 2004. A survey assessing the usefulness of the rankings was sent to all county health officers following its release.

Significant disparities exist in the health outcomes and determinants of Wisconsin counties. We used Pearson product moment correlation and found that, overall, the summary determinant and summary outcome ranks were well correlated (*r* = 0.75). Compared with the healthiest counties (e.g., Ozaukee), the least healthy counties (e.g., Menominee) showed greater improvement in health over time. Of the county health officers participating in the survey of the rankings (N = 68, 94% response rate), 82% reported that the rankings were useful to their work, and 69% planned to use the rankings in their communities. Suggestions received through this survey and other more informal feedback will be incorporated into the 2004 rankings, such as the expansion and improvement of the environmental health components and the inclusion of additional local survey data.

The *Wisconsin County Health Rankings* provides a valuable vehicle for delivering and discussing county-level health information and for engaging stakeholders in the discussion of approaches for reducing observed disparities. This report will continue to be produced annually with special attention given to improving population health measures and its use in community health improvement efforts.

# Sub article: Colorectal Cancer Screening in Washington State: Predictors of Current Screening and Explanations for No Screening

**Published:** 2005-03-15

**Authors:** Peggy A Hannon, Jeffrey Harris, Diane Martin, Juliet VanEenwyk, Deborah Bowen

**Affiliations:** University of Washington

## Track: Social Determinants of Health Inequities

The purpose of this study was to identify predictors of current colorectal cancer screening in Washington State and to examine participants’ reasons for not being screened.

We analyzed data from the 2002 Behavioral Risk Factor Surveillance System for Washington State residents aged 50 years and older (N = 2109). Current colorectal cancer screening was defined as having a fecal occult blood test (FOBT) within the past year and/or sigmoidoscopy or colonoscopy within the past five years. Participants who did not have current FOBT or current endoscopy were asked the primary reason for not obtaining screening.

Overall, current colorectal cancer screening was reported by 51.9% of the sample (FOBT by 25.8%; endoscopy by 42.8%). Univariate analyses showed that several demographic characteristics were significantly associated with screening status, including white race (*P* = .04), aged 65 years or older (*P* < .001), annual income more than $75,000 (*P* < .001), and having a college degree (*P* = .02).

In a multivariate analysis adjusting for the above characteristics and other likely confounding variables (e.g., sex, marital status), participants were significantly more likely to have current screening if they possessed health insurance (54.2% vs 16.8% for uninsured participants, *P* < .001) and had discussed colorectal cancer screening with a health care provider (67.3% vs 33.4% for participants who had never discussed screening with a health care provider, *P* < .001). Participants were also significantly more likely to report current screening if they lived in a large town or urban area (53.0% vs 42.7% for participants living in small towns/rural areas; *P* = .05).

The majority of participants without current screening cited lack of awareness as the primary reason for not being screened (53.0% for FOBT; 46.9% for endoscopy). An additional group of participants stated their physicians had not recommended screening (24.8% for FOBT; 33.3% for endoscopy). Relatively few participants said they were not willing to be screened (20.4% for FOBT; 18.1% for endoscopy) or cited lack of access (1.8% each for FOBT and endoscopy).

Our results indicate that nearly half of age-appropriate Washington State residents lack current colorectal cancer screening. Awareness of colorectal cancer screening, particularly via speaking with a health care provider, was an important predictor of screening. These findings are consistent with published reports based on National Health Interview Survey data. Interventions should be developed to increase awareness of and physician recommendations for colorectal cancer screening, particularly among disadvantaged patient populations.

# Sub article: Educating California School Board Members: Aligning Policies for Student Health and Achievement

**Published:** 2005-03-15

**Authors:** Peggy Agron, Victoria Berends

**Affiliations:** California Department of Health Services, California Project LEAN (Leaders Encouraging Activity and Nutrition)

## Track: Policy and Legal

The objective of this project was to share the development, implementation, and outcomes of a three-year intervention aimed to educate California school board members about the important role school nutrition policies can play to increase the health and academic achievement of students.

California Project LEAN (CPL), the California School Boards Association (CSBA), the University of South Florida, The California Parent Teachers Association, and 10 regional collaboratives worked together to educate school board members across California. Special outreach efforts were directed toward school districts serving low-income children.

Formative research was conducted to understand the factors that influence policy decision making for California school board members and included a literature review, a media analysis, key informant interviews, and a statewide survey of California school board members and superintendents. The formative research served as the foundation for developing a social marketing plan and intervention strategies. Research indicated that CSBA was highly respected by its members. The following activities were implemented jointly by CSBA and CPL: 1) the development of a *Healthy Food Policy Resource Guide (Guide)*; 2) placement of advertisements and articles published in CSBA communications; 3) training of more than 300 school board members across the state; and 4) local mobilization led by CPL regional coordinators.

The evaluation of this three-year project consisted of 1) surveys of school board members who received the guide and attended a training; 2) key informant interviews; and 3) a postsurvey of school board members and superintendents.

Evaluation findings suggested that the *Guide* was useful to school board members and that members who attended trainings planned to raise the issue of nutrition policy for discussion at a school board meeting. A number of California districts have established policies that support healthy eating. Postsurveys and postinterviews were analyzed, and final data was available by January 1, 2005. The impact of this project continues to be realized as more school districts request assistance for mobilizing to offer healthier foods for their students.

The nation is experiencing an unparalleled obesity epidemic. Many of the foods children eat at school are high in fat, sugar, and calories. These practices can contribute to inadequate diets and the development of poor dietary habits. Developing targeted campaigns with respected education partners can provide local policy makers with resources to help them establish policies to support healthy eating.

# Sub article: Adherence to Guidelines for Following Up Low-Grade Pap Test Results by Age and Race or Ethnicity

**Published:** 2005-03-15

**Authors:** Vicki B Benard, Herschel Lawson, Christie Eheman, Christa Anderson, William Helsel

**Affiliations:** Centers for Disease Control and Prevention, National Center for Chronic Disease Prevention and Health Promotion, Division of Cancer Prevention and Control

## Track: Health System Change

The objective of this study was to determine if low-income and uninsured women in the National Breast and Cervical Cancer Early Detection Program (NBCCEDP) with Papanicolaou (Pap) test abnormalities of atypical squamous cells of undetermined significance (ASCUS) or low-grade squamous intraepithelial lesions (LSIL) were followed by the recommended interim guidelines for management of abnormal cervical cytology.

For this study period (July 1991 through September 2000), the National Cancer Institute's (NCI's) recommended guidelines for women with a low-grade abnormality Pap test result (ASCUS or LSIL) was follow-up by Pap tests repeated every four to six months for two years. If a second ASCUS or LSIL report occurred, the patient should have been considered for colposcopic evaluation. We analyzed data from 10,004 women in the NBCCEDP with ASCUS or LSIL followed by a second low-grade abnormality. The racial/ethnic groups included in the analysis were white, black, Asian/Pacific Islander, American Indian/Alaska Native, and Hispanic.

Using recommended guidelines, 44% of women in the NBCCEDP were followed appropriately with a colposcopy following two low-grade abnormalities. Younger women (under 30) were more likely to receive a colposcopy following two low-grade abnormalities, and older women (over 60) were more likely to receive a third Pap test. Hispanic or Latino women were more likely than other racial/ethnic groups to receive a colposcopy after two low-grade abnormalities, and American Indian or Alaska Native women were more likely than other racial/ethnic groups to receive a third Pap test.

Less than half of the women studied were followed by the recommended guidelines. Factors such as age and race/ethnicity influence the appropriate follow-up of a woman with cytological abnormalities. From this study, we are not able to determine if these differences occur at the patient or provider level. However, the national program is working with state, territorial, and tribal programs to further investigate the issue and recommend interventions to improve the level of follow-up.

# Sub article: VERB™ Campaign: Extending the Reach of a National Campaign to Ethnically Diverse Audiences

**Published:** 2005-03-15

**Authors:** Rosemary Bretthauer-Mueller, Heidi Melancon

**Affiliations:** Centers for Disease Control and Prevention, National Center for Chronic Disease Prevention and Health Promotion, Division of Adolescent and School Health

## Track: Communications and Technology

The objective of the Centers for Disease Control and Prevention’s (CDC's) “VERB. It’s what you do.” campaign is to increase and maintain physical activity among “tweens,” or children aged nine to 13 years.

VERB™, a national social marketing campaign with ethnic market overlays, reaches all tweens across the nation with messages designed to get them up and moving. To ensure that all segments of the multicultural audiences are reached by the campaign, the CDC worked with four multicultural advertising/marketing agencies to supplement and complement the general market communication with culturally relevant messages and executions through appropriate channels. The campaign’s efforts extend an invitation to Native American, African American, Asian American, and Hispanic/Latino tweens to take part in the VERB campaign. These culturally and linguistically relevant efforts also help to fill in the gaps inherent in general market communication channels that reach tweens in addition to those that reach parents and other adult influencers. The VERB executions expand campaign messages, reach, impact, and effectiveness.

To reach ethnic audiences, the four multicultural agencies have produced a marketing mix that includes television, radio, out-of-home, and print advertising; in-school promotions; viral marketing; events; and public relations. As is the standard for the VERB campaign, these products were developed on the basis of extensive formative and message-testing research. The culturally relevant products are also strongly rooted in the VERB brand strategy to maintain synergy with the general market efforts, which is critical to a seamless campaign.

The campaign’s national longitudinal evaluation indicates that 63% of African American tweens and 70% of Hispanic/Latino tweens are aware of the VERB brand, exceeding the campaign’s goal of 50% awareness. A special survey was administered in-language with Asian language-speaking parents of tweens living in the Los Angeles area. The results indicate that the parents surveyed were more aware of VERB than any other parental ethnic group.

# Sub article: Racial Differences in Factors That Influence Survival With Oral Cancer in Georgia: 1978–2001

**Published:** 2005-03-15

**Authors:** Ranjitha Krishna, Jonathan Liff, Amy Chen, Paul Eke, Robison Valerie

**Affiliations:** Centers for Disease Control and Prevention, National Center for Chronic Disease Prevention and Health Promotion, Division of Oral Health

## Track: Health System Change

The purpose of this study was to examine the racial differences in distribution of risk factors associated with oral cancer survival in Georgia (1978–2001).

Studies have shown that the five-year survival rate for people with oral cancers is much lower in blacks than whites. According to the Surveillance, Epidemiology and End Results (SEER) program, the national five-year survival rate for people with cancers of the oral cavity and pharynx from 1992 to 1997 was 36.1% for blacks and 59.7% for whites.

Data from 1503 whites and 531 blacks with oral cancers in five urban and 10 rural counties of Georgia from 1978 to 2001 were analyzed. Data were collected by the Georgia Center for Cancer Statistics, a population-based cancer registry affiliated with the SEER program. Racial disparities were examined in stage at diagnosis, grade of cancer, sex, age, socioeconomic status, rural/urban residence, and type of treatment.

Compared with whites, blacks were twice as likely (Odds Ratio [OR] = 2.5, 95% Confidence Interval [CI], 2.0–3.0) to die during the five-year follow-up time. Compared with whites older than 70 years, blacks were 2.2 times (95% CI, 1.6–3.1) more likely to be diagnosed at age 61 to 70 years, 3.7 times (95% CI, 2.7–5.1) more likely to be diagnosed at age 51 to 60 years, and 4.8 times (95% CI, 3.4–6.7) more likely to be diagnosed at younger than 50 years. Compared with whites who were mostly diagnosed at the localized stage of the disease, blacks were 3.0 times (95% CI, 2.4–3.8) more likely to be diagnosed at the regional stage and 4.8 times (95% CI, 3.4–6.7) more likely to be diagnosed after distant metastasis. Blacks were also more likely to have grade 2 (OR 3.0; 95% CI, 2.3–3.9) and grade 3 (OR 2.2; 95% CI, 1.6–3.1) cancers. Consequently, blacks were 95% more likely than whites to have received radiation (OR 3.1; CI, 2.2–4.3) or both radiation and surgery (OR 2.3; 95% CI, 1.7–2.9). Whites were more likely than blacks to have received surgery only.

In Georgia, diagnoses of oral cancers in black patients occurred at a much younger age and at a more advanced stage and higher grade of disease. Oral cancers in black patients were also treated with other than cancer-directed surgery only. These disparities highlight both a challenge to further understand the reasons for racial disparities in survival rates of patients with oral cancers and an opportunity to reduce those disparities.

# Sub article: Eliminating Disparities in Communities of Color Through the Lifetime Fitness Program

**Published:** 2005-03-15

**Authors:** Susan J Snyder, Basia Belza

**Affiliations:** Senior Services of Seattle/King County, Senior Wellness Project

## Track: Evidence-based Programs: Research, Translation, and Evaluation

The objective of this study was to convey lessons learned about factors that contribute to sustainable and effective group fitness programs for older adults in ethnic communities.

The University of Washington Health Promotion Research Center conducted focus groups with older adults from seven cultural groups (American Indian/Alaska Native, African American, Chinese, Korean, Spanish-speaking Latinos, Filipinos, and Vietnamese) to generate ideas for programming that would increase the level of physical activity in these communities. After focus group results were compiled and published, an evidence-based group exercise program for older adults — the Lifetime Fitness Program (LFP) — was implemented in 11 focus-group communities that also had a nutrition program. The 11 communities were located in Texas and western and central Washington.

The LFP was designed by researchers and specialists in aging at the University of Washington in Seattle as an easy-to-implement fitness program aimed directly at older adults. Average age of participants at all LFP sites (N = 3258) is 74.3 years (SD ± 8.7). The program is offered in hourly sessions two to three times per week and includes strength, endurance, balance, and flexibility exercises. LFP Testing of Function for each participant is conducted at enrollment and every four months thereafter.

Focus group findings showed that both the key motivator and primary barrier for physical activity were related to health and chronic conditions. Ideal fitness program components that were common across the groups were programs that included peer support and instruction, were offered in locations close to where attendees lived and in a center that was targeted to their ethnicity, and included several options for exercising (e.g., alone, in a group). Detailed results of these focus groups are published in the report *Elder Perspectives on Physical Activity: A Multicultural Discussion*.

Preliminary data reported here include 226 LFP participants from 11 ethnic sites (average age, 72.8 years; SD ± 8.7). Participants had at least one valid outcomes measure; 27% (n = 62) had four-month follow-up data. At baseline, percentages of participants below normal limits were the following: in arm curls, 23% of participants at ethnic sites and 10% at nonethnic sites; in Up and Go, 68% at ethnic sites, 36% at nonethnic sites; and in chair stands, 30% at ethnic sites and 21% at nonethnic sites. Normal limits were obtained from published age- and sex-based cut points. 

Significant improvement was seen in chair stands and arm curl repetitions at ethnic sites at four months. At follow-up (n = 62), percentages of participants below normal limits were the following: arm curls (2% at ethnic sites, 4% at nonethnic sites), Up and Go (49% at ethnic sites, 29% at nonethnic sites), and chair stands (7% at ethnic sites, 12% at nonethnic sites).

Knowledge gained from these focus groups and from the implementation and evaluation of the LFP can inform future interventions to better reach ethnic minority communities. A policy that links senior nutrition sites serving minority communities to evidenced-based programs such as the LFP may be an effective way to reduce health disparities. Since this abstract, additional data have been collected and analyzed.

# Sub article: Developing a Rural Health Promotion Specialist Program to Provide Preventive Health Care to the Medically Indigent

**Published:** 2005-03-15

**Authors:** Joy N Maltese, Cary Brewton

**Affiliations:** Georgia Department of Human Resources, Division of Public Health, District 4 Health Services

## Track: Partnerships

The objective of this program was to recruit and train individuals to help promote preventive health care in underserved rural areas. The program took place in Troup County and Heard County communities in rural west Georgia.

The District Four Public Health Chronic Disease Prevention Initiative made minor modifications to the Racial and Ethnic Approaches to Community Health (REACH) 2010 program (designed for urban areas) and implemented it in our rural communities. To recruit lay volunteers, we primarily focused on the faith community but also included private businesses such as laundromats and community centers. Volunteers were trained in blood pressure screenings, body mass index (BMI) measuring, diet counseling, exercise, and resources to support individuals screened. A resource library was established with supporting information that included pamphlets and flip charts to help facilitate volunteers’ interaction with individuals at risk. Health promotion specialists collected and submitted contact data for aggregate review to the District Health Services Chronic Disease Prevention staff.

After one-day training was provided to 15 health promotion specialists on February 7, 2004, 134 people were screened over a three-month period between February and June 2004. Of the individuals screened, 98% were African American, lived in rural areas of west Georgia, and were medically indigent or had limited access to health care. The data showed that 39% of the individuals screened were prehypertensive and 37% were either in stage 1 or stage 2 hypertension for their systolic measurement. The BMI measurements showed that 34% were overweight and 40% were considered obese. Overall, 76% of those screened showed hypertensive risks that correlated to 74% that were overweight or obese.

Futhermore, eight individuals who had stage 2 hypertension reported not taking medications as directed by their doctor. The health promotion specialist was able to encourage these people to resume their medications and recorded a return to normal blood pressures usually after two to four weeks of taking medications on a regular basis.

Having lay volunteers trained as health promotion specialists in rural areas is critical in preventing stroke and heart attack and reducing unnecessary emergency department visits in the absence of a health care provider. Individuals screened and counseled responded well to the advice given by people they know and trust in their churches, community centers, and local businesses. We currently have a waiting list for people to be trained and hope to expand this initiative to every community in our health district and, eventually, throughout Georgia.

# Sub article: Regional/Racial Prevalence of Metabolic Syndrome: The MSM Regional Assessment Health Surveillance Study, 2003–2004

**Published:** 2005-03-15

**Authors:** Cassandra Arroyo, Dennis Jones, Yong Liu, Rebecca Din-Dzietham, Sharon Davis

**Affiliations:** Morehouse School of Medicine, Social Epidemiology Research Center

## Track: Evidence-based Programs: Research, Translation, and Evaluation

The objective of this study was to examine regional and racial variations in the prevalence of metabolic syndrome (MetS) in Fulton, Bulloch, Candler, Evans, and Jenkins counties of Georgia.

Random-digit–dialing data followed by examination data were obtained from 319 African American and white men and women aged 19 years and older from 2002 through 2003. MetS was defined by Adult Treatment Panel III criteria. Correlates included race (African American vs non-Hispanic white), sex, education level, age, and region (urban vs rural). Univariate and multiple regression models were used to assess the interaction between region and race, and the association with correlates setting nominal P value at .05 for main effect and .10 for interaction. SUDAAN (Research Triangle Institute, Triangle Park, NC) was used to account for the complex design and to obtain correct variance and county-representative estimates.

The MetS overall prevalence was 21.2%. Unadjusted prevalence of MetS was significantly higher (P < .001) in urban areas (21.4%) vs rural areas (19.6%) among African Americans (31.1%) vs non-Hispanic whites (9.6%) and among women (22.2%) vs men (19.9%). There was a significant interaction between region and race (P < .001), so separate models were estimated for African Americans and non-Hispanic whites. For African Americans, MetS was 2.47 (95% Confidence Interval [CI], 2.23–2.73) times more prevalent among those living in urban vs rural areas and 0.48 (CI, 0.46–0.50) times less prevalent among men vs women. Prevalence of MetS was also 1.48 (CI, 1.40–1.56) times higher among those with less than 12 years of education and 0.68 (CI, 0.65–0.72) times lower among those with 12 years of education vs those with more than 12 years. Among non-Hispanic whites, MetS was 0.34 (CI, 0.32–0.37) times less prevalent among those living in the urban area, 6.13 (CI, 5.60-6.71) times more prevalent among men, 7.9 (CI, 7.12–8.68) times more prevalent among those with 12 years of education, and 4.6 (CI, 3.82–5.66) times more prevalent among those with less than 12 years of education.

The study suggests that African Americans living in the urban area of Georgia have a higher prevalence of MetS than their white counterparts. National prevalence rate estimates for MetS suggest that whites in general have a higher prevalence of MetS. A more comprehensive database is needed to further explore this interaction between race and region to target more specific groups for intervention.

# Sub article: A Community-based Partnership to Address Barriers to Physical Activity in an African American Community

**Published:** 2005-03-15

**Authors:** Marcus Plescia, Martha Groblewski

**Affiliations:** North Carolina Division of Public Health, Chronic Disease and Injury Section

## Track: Partnerships

The objective of this project was to describe changes in physical activity behaviors resulting from a partnership between a community coalition, lay health advisors, and a YMCA branch.

The Centers for Disease Control and Prevention’s Racial and Ethnic Approaches to Community Health (REACH 2010) project funds 37 communities to engage in participatory, community-based interventions to address racial and ethnic health disparities. Our project targets a geographically defined, urban, medically underserved, African American community of 18,892 in Charlotte, NC.

The focus of the project is to recruit, train, and support lay health advisors to promote health behavior change among community residents. A partnership with a local branch of the YMCA was designed to support the efforts of the lay health advisors and address existing barriers to participation in regular physical activity. Residents are encouraged to participate in community-based YMCA activities that occur in a range of settings and are funded by the REACH 2010 coalition. Evaluation methods include 1) a yearly random survey of community residents by telephone using Behavioral Risk Factor Surveillance System questions on physical activity behaviors; 2) pretest and two-year follow-up surveys by telephone of program participants to measure relative frequency of physical activity and current stage of change for physical activity behavior change; and 3) focus groups to determine motivational factors among community participants in the YMCA program.

The project has maintained an average of 15 active lay health advisors working at least 10 hours per week in the community. In the second year of the project, the percentage of adults in the focus community who indicated they did not meet physical activity recommendations decreased from 32.0% (95% Confidence Interval [CI], 28.6–35.5) at baseline to 24.4% (95% CI, 21.1–28.2). At two-year follow-up of YMCA program participants, 56.6% indicated that they were exercising more than they did two years before, and the percentage who indicated Maintenance Stage of Change increased from 41.67% (95% CI, 37.31–46.03) to 54.24% (95% CI, 48.01–58.65). Focus group respondents identified intrapersonal factors such as improved self-efficacy and interpersonal factors such as social support and fellowship as aspects of the YMCA programs that helped motivate them to continue participation in the program.

Partnership between a REACH 2010 community coalition and a local YMCA branch was instrumental in establishing and supporting a community lay health advisor program and addressing community barriers to physical activity. This multicomponent intervention resulted in significant physical activity behavior change at the individual and community levels.

# Sub article: Getting the Most Out of Vital Statistics Data: Diabetes-related Heart Disease Mortality in New Mexico

**Published:** 2005-03-15

**Authors:** R. Heidi Krapfl, Dorothy Gohdes, Janet Croft

**Affiliations:** New Mexico Department of Health, Diabetes Prevention and Control Program

## Track: Methods and Surveillance

The objective of this study was to illustrate how multiple-cause mortality data can enhance interpretation of heart disease (HD) mortality among racial/ethnic groups.

Multiple-cause mortality files for New Mexico from 1999–2001 were obtained from the National Center for Health Statistics at the Centers for Disease Control and Prevention. Deaths from HD for New Mexico residents were identified by the International Classification of Diseases, Tenth Revision (ICD-10) codes I00–I09, I11, I13, and I20–I51. Premature heart disease (PHD) was defined as any underlying HD death occurring in persons aged less than 65 years. Diabetes-related HD was classified as any death where the underlying cause of death was HD, and diabetes (ICD-10 codes E10–E14) was reported as any of up to 20 contributing causes of death. Residents were grouped into four racial/ethnic categories: non-Hispanic white, Hispanic of any race, non-Hispanic American Indian, and other. All death rates for HD were calculated with bridged-race population estimates and age-adjusted to the 2000 U.S. Standard Population.

From 1999 to 2001, 24% of all deaths in New Mexico reported HD as the leading cause of death. Of these deaths, 16.6% occurred in persons aged less than 65 years and were therefore classified as premature. The proportion of PHD deaths was substantially higher in the American Indian (29.2%) and Hispanic (20.8%) populations than in whites (13.7%). Diabetes contributed to almost 18% of PHD deaths in American Indians and Hispanics and to 10% of PHD deaths among whites.

Multiple-cause mortality data indicate that the contribution of diabetes to PHD is disparate among racial/ethnic groups in New Mexico. These results support continued analysis of these data in a consistent manner and further underscore the growing threat of diabetes to communities in the United States. Much of the progress in decreasing cardiovascular disease in the United States may be lost as increasing diabetes and obesity lead to PHD death in many populations.

# Sub article: Overcoming Barriers in Access to Specialized Health Care Services

**Published:** 2005-03-15

**Authors:** Donna Russell Doran, Jeffrey Gold, Francis Serbaroli, Edward L Hannan, Paul F Macielak, William F Streck

**Affiliations:** New York State Department of Health, Cardiac Service Program

## Track: Policy and Legal

The objective of this interdisciplinary statewide initiative was to identify factors limiting access to specialty care and to develop strategies to overcome these barriers. We focused on barriers that are amenable to resolution through health care policy, regulation, or statute and on innovative strategies that could be implemented within the resource constraints of our health care system.

A Quality Initiatives Workgroup (QIW) of the New York State Public Health Council (PHC) was convened to examine issues and potential resolutions associated with disparities in access to specialty care for New York State residents. The work group was composed of members of the PHC and experts from pertinent fields. In addition to gathering input from QIW panel members on clinical, analytical, and financial issues, the group set out to gather information through roundtable discussions with a broad range of experts (including regulators, insurers, providers, researchers, patient advocates, medical educators, and professional societies), literature reviews, and responses from a survey aimed at gaining a broad perspective on barriers and potential solutions to accessing specialty care.

The QIW’s review of expert testimony, questionnaire responses, and research concluded that disparities in access to care exist and that these disparities have a significant and costly impact on the overall health of New York State residents. Factors associated with barriers to appropriate health care fall into the following categories: health care delivery issues (including provider awareness and implementation, patient-provider communications, and system navigation issues), availability of services, insurance coverage, and applied research and quality initiatives. Specific issues, goals, and recommended actions are identified in each of these areas. Sufficient evidence is available to support implementation of well-planned strategies aimed at reducing existing inequities. In evaluating strategies for overcoming barriers, the QIW recommends an initial focus on seven clinical areas: heart disease, stroke, cancer, major orthopedic conditions, diabetes, end-stage renal disease, and HIV/AIDS. These diseases impact a substantial portion of the population and have relatively mature and accepted clinical treatment protocols (thus making them amenable to monitoring). Additionally, inequities to care have been identified in the literature for these diseases.

Recommended actions — some of which are being implemented — will be discussed. These recommendations will provide a stimulus, foundation, and preliminary framework for groups to build upon; offer guidance for evaluating the provision of specialized services; identify challenges that cut across the health care sector; and provide a road map for remediation.

# Sub article: Reducing Dental Sealant Disparities in School-aged Children Through Better Targeting of Informational Campaigns

**Published:** 2005-03-15

**Authors:** Kari A Jones, Susan Griffin, Ramal Moonesinghe, Freder Jaramillo, Claudia Vousden

**Affiliations:** Centers for Disease Control and Prevention, Division of Public Private Partnerships

## Track: Communications and Technology

The objective of this study was to investigate whether disparities in receipt of dental sealants among school-aged children are linked to caregivers’ knowledge of the preventive purpose of sealants. These findings may be used to better target oral health education campaigns.

Using data from the National Health and Nutrition Examination Survey (NHANES) 1999–2000, we estimated sealant prevalence among children aged six to 17 years who had at least one tooth eligible for a sealant. We then identified the explanatory factors (main effects model) associated with knowledge of sealants among caregivers of children aged less than 18 years using data from the 2003 ConsumerStyles, HealthStyles, and Recontact marketing surveys and logistic regression. We stratified the data from the marketing surveys on sealant knowledge and the NHANES data on sealant prevalence by race/ethnicity and income (whether ⩾ or < 200% of the 2003 federal poverty guidelines), the two significant explanatory factors common to both data sets (P < .001 for race/ethnicity and P = .01 for income).

Over the full study sample, sealant knowledge was 62.5%, and sealant prevalence was 31%. Caregivers’ race/ethnicity, age, marital status, education, income, and sex were significant predictors of sealant knowledge. Both sealant knowledge and prevalence were positively associated with income level. Among higher-income families, 71% of caregivers exhibited sealant knowledge compared with 47% of their low-income counterparts; 42% of higher-income children had sealants compared with 22% of their low-income counterparts. Among higher-income families, sealant prevalence among children was positively associated with caregiver knowledge (r = 0.973). Non-Hispanic whites had the highest caregiver knowledge (78%) and highest sealant prevalence (49%) in this group; non-Hispanic blacks had the lowest caregiver knowledge (41%) and sealant prevalence (22%). Among low-income families, there was no association between caregiver knowledge and sealant prevalence.

Current sealant prevalence is well below the Healthy People 2010 objective of 50%. We found disparities in both knowledge and prevalence of sealants by race/ethnicity and income. The positive association between sealant knowledge and prevalence for higher-income families is consistent with the economic principle that demand for sealants increases with knowledge of their benefit. The lack of an association between sealant prevalence and knowledge among low-income families may reflect higher levels of public provision of sealants to this group. This suggests that informational campaigns could increase demand for sealants in both income groups. Additionally, efficient targeting — targeting groups with the lowest demonstrated knowledge — should also help eliminate disparities. This information is useful to oral health coalitions funded by the Centers for Disease Control and Prevention in many states to promote oral health and eliminate oral health disparities.

# Sub article: Engaging Older Adults to Be More Active Where They Live: Audit Tool Development

**Published:** 2005-03-15

**Authors:** Judy Kruger, Melissa Kealey, Rebecca Hunter, Susan Ivey, William Satariano, Constance Bayles, Laura Brennan Ramirez, Lucinda Bryant, Courtney Johnson, Chanam Lee, David Levinger, Kathleen McTigue, Gwen Moni, Anne Vernez Moudon, Delores Pluto, Thomas Prohaska, Christen Sible, Sabrina Tindal, Sara Wilcox, Kendra Winters, Kathy Williams

**Affiliations:** Centers for Disease Control and Prevention, National Center for Chronic Disease Prevention and Health Promotion

## Track: Social Determinants of Health Inequities

Because of an increase in the prevalence of chronic diseases and an aging population, older adults are at highest risk for poor health. Health disparities by age and socioeconomic status have been shown to exist for many physical activity outcomes and may be explained in part by differences in the built environment in which older adults live, work, and play. Partners of the Environmental Workgroup of the Centers for Disease Control and Prevention Healthy Aging Research Network (HAN) have designed and piloted an instrument that focuses on the relationship between the built environment and physical activity in older adults.

Neighborhoods in seven diverse U.S. communities served by HAN were studied: Alamosa, Colo; Columbia, SC; Hendersonville, NC; Seattle, Wash; McKeesport, Pa; Chicago, Ill; and Berkeley, Calif. An environmental audit instrument, developed to be sensitive to issues of importance to older adults, was designed to assess street-scale factors associated with physical activity across multiple settings. Audit items included land use, destinations, sidewalk and intersection conditions, amenities such as benches, social disorder such as litter, and the types of human activity observed. In the pilot study, 15–30 street segments were audited by two or more trained researchers at each of the seven HAN sites. In addition to the audit, qualitative semi-structured interviews were conducted to identify additional items of importance to adults aged 65 years or older.

Inter-rater reliability among the trained researchers was examined. Researchers across the seven HAN sites examined differences in neighborhood segments audited to determine the distribution of study variables, such as presence of sidewalks and crosswalks. Qualitative interviews of older adults about environmental influences on walking behavior revealed that having destinations to walk to appeared to be an important motivator for walking, although many people reported both walking to reach destinations as well as taking walks just for pleasure. Destinations such as grocery stores, banks, pharmacies, restaurants, and beauty parlors were mentioned, as were churches, libraries, parks, and the homes of family, friends, and neighbors. Personal safety was another frequently mentioned topic, with many residents feeling safe walking during the daytime but not at night. Interviewees indicated that their choice of walking routes was influenced by the length of the route, sidewalk quality, people along the route, amount of traffic, crosswalks and signals for crossing the street, perceived safety from crime, scenery, aesthetics, and presence of interesting things to look at. Future directions include using the environmental audit instrument in a study to see which environmental audit items are correlated with actual walking behavior in an older population.

# Sub article: Conducting a Successful “Through With Chew” Week

**Published:** 2005-03-15

**Authors:** Sylvia Bagdonas, Niki Mueller, Amy Russian

**Affiliations:** Wyoming Department of Health, Substance Abuse Division

## Track: Communications and Technology

An idea of the American Academy of Otolaryngology — Head and Neck Surgery, “Through With Chew” Week is an educational campaign designed to prevent and reduce the use of chew/spit tobacco, a type of tobacco use that has not received as much attention as cigarette smoking. The state of Wyoming successfully conducted a “Through With Chew” Week (including a Great American Spit Out Day) in February 2003. The statewide program targeted Wyoming’s small and rural population, which has a disproportionately high chew-tobacco-use rate — the second highest in the nation (Behavioral Risk Factor Surveillance System [BRFSS], Adult Tobacco Survey [ATS], 2003).

The component aims of the program were the following: 1) to educate the public about the extent of chew-tobacco use in Wyoming and its costly health implications for all citizens; 2) to conduct surveys to establish baseline data on current levels of prevention and intervention; 3) to partner with health care providers to promote quitting; 4) to counteradvertise the tobacco industry; and 5) to determine adaptations necessary for American Indian populations. Wyoming’s comprehensive statewide plan included tool kits, media kits, and “quit spit” kits for use by local program managers. Extensive media coverage included newspaper and pizza-box ads, television coverage, and presentations by Gruen Von Behrens, a 25-year-old man severely marred by oral cancer. Additional “guerrilla” advertising was conducted in barbershops, rodeos, fairgrounds, little league fields, bowling alleys, agriculture shops, and publications.

Follow-up surveys to Tobacco-Free Wyoming Communities and the dental community indicated that dental office interventions increased by 58%, and response volumes to Wyoming’s Quitline and QuitNet from chew tobacco users doubled. Wyoming’s “Through With Chew” campaign, including lessons learned and tangible tools for success, can be replicated easily in other states and communities.

# Sub article: Retooling an Osteoporosis Prevention Program to Serve Low-Income Populations: *A Practical Guide to Bone Health*

**Published:** 2005-03-15

**Authors:** Denise R Cyzman, Norma Bour, Rachel McLaury, Judith Lyles

**Affiliations:** Diabetes, Kidney, and Other Chronic Diseases, Michigan Department of Community Health

## Track: Partnerships

We modified an existing osteoporosis prevention program to address the needs of low-income consumers in Michigan and conducted a pilot test to assess the program’s effectiveness.

Building on previously collected focus group data from consumers and educators, we surveyed eight educators with the Michigan State University Extension (MSUE) to help identify barriers encountered delivering an educational osteoporosis prevention program to low-income constituents. The educators recommended program content changes that placed more emphasis on practical information relating to food choices, nutrition, and physical activity. Registered dietitians and staff representing the Michigan Nutrition Network, United Dairy Industry, and the Michigan Department of Community Health developed new material. The resulting program, A Practical Guide to Bone Health, was designed as a 45-minute presentation available in either a flip-chart or PowerPoint format. The content emphasized physical activity, diet quality, and the importance of calcium and vitamin D. A shopping section guided consumers to better food choices. The program also encouraged change in diet and physical activities. A statewide steering committee reviewed the program before a final field test.

We conducted the program at four sites, one urban and three rural, selected by MSUE educators who reach low-income populations. Participants completed pre-tests and post-tests assessing basic knowledge and intent to change behavior. Educators documented program location, number of participants, and audience income level.

A total of 46 people participated in the program. Participants were primarily between the ages of 18 and 44 years (75%), white (93%), and female (79%); at three of four sites, participants were predominantly low-income. A total of 43 participants completed both the pre-test and the post-test (93%). Of those who did not have perfect scores on the knowledge pretest, 73% increased their scores following the session. Prior to the program, 28% reported inadequate calcium intake and 30% reported inadequate physical activity. At post-test, 50% of those with low calcium intake indicated intent to eat three or more servings of calcium-rich foods. Likewise, 62% of those with low activity levels reported intent to increase physical activity to at least 30 minutes three times a week. An additional 13% indicated intent to increase activity to 30 minutes five times a week.

Collaboration among public health practitioners resulted in an easy-to-use and effective osteoporosis prevention program directed toward the needs of low-income populations.

# Sub article: Making the Grade on Women’s Health: A National and State-by-State Report Card 2004

**Published:** 2005-03-15

**Authors:** Michelle Berlin, Judith Waxman

**Affiliations:** OHSU National Center of Excellence in Women's Health, and Associate Professor, Departments of Obstetrics and Gynecology, Public Health and Preventive Medicine, and Medical Informatics and Clinical Epidemiology, Oregon Health and Sciences University

## Track: Methods and Surveillance

*Making the Grade on Women's Health: A National and State-by-State Report Card* is a comprehensive study of the status of women's health in the United States based on selected health status and policy indicators. Health status indicators reflect conditions with a significant impact on quality of life and well-being, affect large numbers of women generally or disproportionately affect a specific population and/or age group, are amenable to prevention or improvement, and are measurable through consistent, reliable data. The 27 indicators fall within four categories: women's access to health care services, preventive health care activities, key women's health conditions, and whether women live in healthy communities.

The states and the nation received grades for each status indicator  based on whether the benchmark was met (Satisfactory) or not met (Satisfactory Minus, Unsatisfactory, or Fail, based on scores' distance from the benchmark). In the 2004 *Report Card*, grades take into account that states and the nation have several years to achieve those benchmarks based upon *Healthy People 2010*. The *Report Card* also provides 67 policy indicators to evaluate the performance of state and federal governments in promoting women's health; these are based on state statutes, regulations, and policies and programs that address problems identified by health status indicators.

The nation met only two indicators (mammograms in women aged >40 years and annual dental visits) and received an overall grade of Unsatisfactory. All states and the District of Columbia met one benchmark (annual dental visits) and missed eight (including proportion of women with health insurance, rates of high blood pressure and diabetes, infant mortality, poverty, and wage gap). Twenty-five states improved at least five policies, and the majority of states weakened in one to three policies. The policy most consistently improved was preventing tobacco sales to minors. Only one policy goal, Medicaid coverage for breast and cervical cancer treatment, was met by all the states.

For most health status indicators, the nation and the states fall short of meeting national goals. Despite interest in health disparities, key differences (including race/ethnicity and age) persist. A number of state governments improved policies in key areas to meet women's health needs, but many states have fallen behind. The 2004 *Report Card* findings identify pressing issues that must be tackled by policy makers, public health administrators, and care providers. To improve and maintain the health of U.S. women, these issues must be addressed swiftly and accurately.

# Sub article: Response Shift: The Measurable and Desired Outcome of Chronic Disease Self-management Programs That Violates Pre–Post Assessment

**Published:** 2005-03-15

**Authors:** Richard H Osborne, Melanie Hawkins

**Affiliations:** The University of Melbourne Department of Medicine, Centre for Rheumatic Diseases, Royal Melbourne Hospital

## Track: Evidence-based Programs: Research, Translation, and Evaluation

Chronic disease self-management programs are designed not only to empower participants and increase their knowledge but also to provide participants with existential insights into their health and ability to self manage. The outcome of a self-management program is traditionally evaluated by comparing pre-intervention and post-intervention questionnaires, a methodology that assumes that participants answer questions from the same perspective before and after the program. Systematic reviews of the outcomes from self-management interventions that use self-appraisal outcomes identify small to no effects. This study of community-based self-management programs in Australia aimed to determine if changes in internal values or perspectives (termed a response shift) occurred in participants and if *response shift* is measurable with a paper-based questionnaire.

The HEI-Q-Perspective Questionnaire, a retrospective nine-item post-test questionnaire, was developed to measure potential benefits of self-management programs. Cognitive interviews with respondents elicited spontaneous statements about the reasons for their paper-based answers across the nine items. The sensitivity, specificity, and overall accuracy of the questionnaire were calculated using the interview as the gold standard. A response shift can be negative (i.e., after the course, participants report they now realize that before the course they were worse than they thought they were), positive (i.e., participants now realize they were better before the course than they thought they were), or neutral (no change).

In-depth interviews (n = 39) and mailed questionnaires (n = 132) reflected that a “true” response shift occurred in about half of the questionnaire items. Of these, 33% had a negative response shift, 18% had a positive response shift, and approximately 32% had no response shift. The presence or absence of response shift could not be determined in approximately 17% of cases. Substantial concordance between interview and questionnaire were observed (average overall accuracy 0.79), indicating the questionnaire effectively identified response shift. A positive or negative response shift was found to have profound effects on patient-reported outcomes — even large positive or large negative program effects revealed in an interview could be concealed in an individual’s pre–post score. This clearly demonstrates that response shift can violate classic outcome assessment of self-management programs.

Response shift occurred in about half of the participants. This result suggests that classic outcome assessment (pre-test vs post-test) in many individuals is flawed. Response shift is a desirable outcome of courses but has not been formally measured. The strong concordance between the questionnaire and cognitive interviews indicates the HEI-Q-Perspective can detect response shift. This new questionnaire will assist researchers and program evaluators to better estimate the impact of self-management programs and to understand the role of response shift in this and other settings.

# Sub article: Moving People to Move: Midpoint Results of the Walk the Ozarks to Wellness Project

**Published:** 2005-03-15

**Authors:** Nikki M Caito, Michael Elliott, Sarah Lovegreen, Paula Klump, Matthew W Kreuter, Ross Brownson

**Affiliations:** Saint Louis University School of Public Health, Health Communication Research Laboratory

## Track: Communications and Technology

The Walk the Ozarks to Wellness Project is a four-year longitudinal study of walking currently underway in six rural underserved communities in southeast Missouri. The project's objective is to increase time spent walking among low-income, overweight, rural Missourians who have diabetes or who are at risk for developing the disease.

Participants initially enrolled in this study at community health events sponsored by a local steering committee. Throughout the first year of the intervention, beginning in November 2003, participants received a monthly newsletter. In the second year, participants receive a bimonthly newsletter. Newsletter messages were written based on participant surveys completed at baseline and at month nine. Topics addressed in the first nine newsletters included motivation, health history, discussions with doctors, self-efficacy, and barriers. Additional newsletters included topics about social support and physical activity level.

To date, 1065 participants have enrolled in the project in nine groups. We have received midpoint (T2) surveys from 153 participants in the first group. Paired t-test analyses show significant improvement at T2 in those who reported no walking and no moderate activities at baseline, in both days per week and minutes per week (P < .001). Of those who marked having no place to exercise as a barrier at baseline, 81% no longer had this barrier at T2. McNemar tests show significant improvement at T2 in those who had not talked to their doctors about healthy eating (P < .001), exercise (P = .029), and losing weight (P < .001) at baseline. Stages of Change analyses show advancement in those who were in the precontemplation and contemplation stages, where overall, 69% in precontemplation and 67% in contemplation moved forward.

These results show the preliminary impact of a tailored intervention in a high-risk population. This tailored intervention was effective in 1) moving participants forward in the initial stages of change; 2) increasing awareness about the importance of talking with one’s doctor about leading a healthy lifestyle; and 3) decreasing perceived barriers.

# Sub article: Colorectal Cancer Screening Among Latinos From U.S. Cities Along the Texas–Mexico Border: A Qualitative Study

**Published:** 2005-03-15

**Authors:** Maria E Fernandez, Isabel Torres, Sally Vernon, Theresa Byrd, Marilyn Hinjosa-Lindsey, Rosario Wippold, Yadvindera Bains

**Affiliations:** University of Texas Health Science Center at Houston School of Public Health, Center for Health Promotion and Prevention Research

## Track: Evidence-based Programs: Research, Translation, and Evaluation

The purpose of this study was to identify factors influencing the decision to undergo colorectal cancer screening among low-income Hispanics living along the Texas–Mexico border. Although colorectal cancer is a leading cause of cancer death among Latinos, most are not getting the recommended colorectal cancer screening (CRCS) tests, and little is known about what types of factors may influence informed decision making for CRCS among Latinos.

Four focus groups with low-income Latino men and women were conducted in January 2004 at each of three sites (Brownsville, El Paso, and Laredo, Tex). Demographic, psychosocial, and cultural factors potentially related to CRCS were addressed, as well as general issues such as access to health care services, perceptions about the importance of preventive health care, and factors surrounding health care decision making.

Both women and men in this study reported a heavy reliance on home remedies, herbal remedies, and prescription drugs bought across the border, in part because of financial barriers and lack of insurance. Many participants in all groups reported feeling more satisfied with the care they received on the Mexican side of the border because of lower-priced medications and office visits and perceptions that care in Mexico is more efficient, flexible, thorough, humane, and because it is offered using the Spanish language. The participants’ knowledge, attitudes, and beliefs related to cancer reflected a mixture of misconceptions and accurate information, and participants expressed varying beliefs about the survivability of cancer. Participants often associated the word *cancer* with descriptors such as death, fear, pain, ugliness, sadness, and hopelessness.

Most participants in this study knew very little about colorectal cancer and even less about CRCS. Many of them were confused about the differences between colorectal cancer and stomach or prostate cancers. There was also a strong belief that untreated hemorrhoids and constipation were major causes of colorectal cancer. Individual-level barriers to CRCS suggested by participants included embarrassment, machismo, lack of knowledge and information, procrastination, fear of questioning physicians, fear of the actual screening procedures, and fear of receiving a diagnosis of cancer. Other barriers to CRCS identified by participants included lack of health insurance or financial resources, being undocumented, and transportation barriers.

Results from these focus groups have provided much-needed preliminary information about this area of Latino health and will provide guidance for the development of interventions to increase CRCS among low-income Hispanics along the Texas–Mexico border.

# Sub article: Challenges to Measuring Health Care Disparities in the *National Healthcare Disparities Report:* Disparities in Data

**Published:** 2005-03-15

**Authors:** Ernest Moy, Irma Arispe, Julia Holmes

**Affiliations:** Agency for Healthcare Research and Quality, Center for Quality Improvement and Patient Safety

## Track: Methods and Surveillance

The objective of this study was to assess the ability of extant national data sets to measure health care disparities in access, use, and quality for different racial and ethnic groups, based on our experiences in developing the Congressionally mandated *National Healthcare Disparities Report* (*NHDR*).

For each of the health care measures included in the *NHDR*, we examined the ability of national data sources to provide information for different groups. We focused on groups specified by 1997 Office of Management and Budget (OMB) Standards: racial minorities, including single-race blacks, Asians, Native Hawaiians and Other Pacific Islanders (NHOPI), American Indians and Alaska Natives (AI/AN), multiple-race individuals, and ethnic minorities (Hispanics).

Measurement challenges were categorized as issues of collection (if data for a particular group were not collected and usable); estimation (if data for a group were collected but suppressed because of small cell size or large relative standard error); and power (if data for a group were collected and adequate to generate estimates but lacked sufficient power to detect relative differences compared with comparison groups of 10% with *P* < .05).

For almost every *NHDR* measure, measurement challenges limited our ability to assess disparities for at least one group. Major measurement challenges varied among groups. Collection issues prevented assessments of disparities for NHOPI and for multiple-race individuals for more than 60% of *NHDR* measures. Estimation issues prevented an assessment of disparities for AI/AN for almost half of measures. Issues of statistical power were common among Asians, NHOPI, AI/AN, and multiple-race individuals. Measures that focus on subsets of the general populations (i.e., women, children, elderly) were particularly vulnerable to measurement challenges.

The goal of reducing disparities in health care depends upon our capacity to measure and track differences in care. For some racial and ethnic minority groups, extant national data are sufficient for assessing many areas of disparity. However, for smaller groups, challenges related to data collection, estimation, and power severely limit our ability to assess disparities. These disparities in data must be addressed to allow design of interventions that reduce disparities in care for all minority groups rather than just the larger groups.

# Sub article: An Alternative to Identifying and Engaging the Underserved, Out-of-Care, HIV High-Risk Population

**Published:** 2005-03-15

**Authors:** Erum Khan Hadi, Sabrina Jaar-Marzouka, Trina Hiemke, Lisa Cardinale

**Affiliations:** Dutchess County Department of Health

## Track: Health System Change

The objective of this project was to provide and assess the effectiveness of a mobile screening and referral program for the medically underserved, high-risk, and difficult-to-reach population of Dutchess County, New York. By U.S. Census 2000 population estimates, 80% of the population of Dutchess County is white, 9.3% is African American, and 6.4% is Hispanic. However, African Americans are 15 times more likely and Hispanics almost 11 times more likely than whites to have HIV. We used an outreach van for HIV screening and referrals at nontraditional service hours and at high-risk venues in two low-income neighborhoods with the greatest burden of HIV disease.

Mobile van outreach workers surveyed each individual screened, and results were reviewed to assess the needs of the HIV-positive out-of-care clients. Outreach service forms and screening forms were analyzed to quantify the number of clients served. Lastly, follow-up phone calls to designated service providers verified whether clients referred made medical visits.

In 2003, the outreach van screened 179 individuals and identified 35 (19.5%) HIV-positive individuals. Of the 35 HIV-positive individuals, outreach workers linked 22 (62.8%) to primary care. Of these 22, 17 (77.3%) were African American and 5 (22.7%) were Hispanic. Dutchess County has an estimated 657 HIV/AIDS-positive individuals out of care; the outreach van identified and provided services to 5.3% (35/657) of this population.

Two of the biggest challenges the program faces are the need to contact high volumes of people to identify the individuals with HIV infection and the issue of safety of the outreach staff and security of the van in high-risk neighborhoods. The success of the mobile screening program can be attributed to the following factors: 1) basic primary care services are brought to the client; 2) the outreach van is staffed with racially diverse peers, outreach workers, and a nurse; 3) the program does not label the van as an HIV-care provider only but provides various other low-threshold screenings, education, and support services; and 4) over time, trusted community leaders referred others in the community to use the van services.

This initial evaluation indicates that the mobile van outreach program is successful in keeping the minority, out-of-care population in underserved neighborhoods engaged in primary care. In addition, the outreach van provides an opportunity for surveillance of HIV and the general health status of high-risk communities; it also increases access to primary care through unique partnerships among service providers.

# Sub article: Is Food Insecurity a Price of Smoking Among the Poor?

**Published:** 2005-03-15

**Authors:** Brian Armour, M. Melinda Pitts, Chung-won Lee, Trevor Woollery, Ralph Caraballo

**Affiliations:** Centers for Disease Control and Prevention, National Center for Chronic Disease Prevention and Health Promotion, Office on Smoking and Health

## Track: Social Determinants of Health Inequities

The objective of this study was to estimate the share of income that families spend on cigarettes and to determine the association between food insecurity, smoking, and poverty.

Cigarette smoking prevalence is higher among adults living below the poverty level. The opportunity cost of smoking (the goods and services smokers forgo to purchase cigarettes) will be proportionately higher among poor families than nonpoor families since a larger share of their income will go toward the purchase of cigarettes. No study has documented what these opportunity costs might be. This study shows an association between food insecurity and the share of income spent on cigarettes. Evidence supporting this association suggests that state-sponsored smoking cessation programs targeting poor smokers may have an added benefit of reducing food insecurity.

Data from the 2001 Panel Study of Income Dynamics (PSID) were used to identify smokers and families that were food insecure and to determine the share of families’ income spent on cigarettes. The PSID is a nationally representative longitudinal study of U.S. families that collects economic, health, and social behavior data on 7406 families. T tests and chi-square tests were used to assess univariate differences between continuous and categorical variables. Multivariate logistic regression models were used to assess the association between food insecurity, smoking, and poverty.

Approximately 7.6% of families lived in poverty, 6.1% were food insecure, and 26.1% had at least one family member who smoked in 2001. Poor families were more likely to have a family member who smoked cigarettes than nonpoor families (33.3%, poor families vs 22.5%, nonpoor families; P < .001). The share of income spent on cigarettes was significantly higher for poor families than nonpoor families (12.0%, poor families vs 2.0%, nonpoor families; P < .001). Results from the multivariate logistic regression analysis revealed that the odds of being food insecure increased as share of income spent on cigarettes increased (adjusted odds ratio, 1.72; 95% confidence interval, 1.66–1.79).

Having an average annual income of $8624 and average annual cigarette expenditures of $857, poor families with a family member who smokes spend a large share of their income on cigarettes. This study suggests that in addition to the adverse health consequences linked to smoking, poor families may also pay a price of food insecurity.

# Sub article: The Incidence of End-Stage Renal Disease in Georgia, 1999–2002

**Published:** 2005-03-15

**Authors:** Karon Abe, Kristen Mertz, Kenneth Powell, Manxia Wu, Pyone Cho

**Affiliations:** Georgia Department of Human Resources, Division of Public Health

## Track: Methods and Surveillance

Each year in the United States, approximately 80,000 people are diagnosed with end-stage renal disease (ESRD), a condition requiring dialysis or kidney transplant to sustain life. The primary causes of the disease for the majority of patients are diabetes and hypertension. We sought to assess racial disparities in the burden of ESRD and its contributing causes in Georgia.

ESRD Network 6 is part of the United States Renal Data System, a nationwide 18-network ESRD surveillance system that collects information on newly diagnosed and chronic ESRD patients. We used data from the ESRD Network 6 Web site to calculate age-adjusted ESRD incidence rates in Georgia and to describe the demographic characteristics of newly diagnosed patients from 1999 through 2002. We also used data from the Behavioral Risk Factor Surveillance System to compare the age-adjusted prevalence of diabetes (2002) and hypertension (2001) among blacks and whites aged 18 years or older in Georgia.

Each year, more than 3000 persons in Georgia are diagnosed with ESRD. From 1999 through 2002, the age-adjusted incidence rate for ESRD was higher in Georgia (42 per 100,000) than in the nation (33 per 100,000). Of the newly diagnosed ESRD patients in Georgia, 57% were older than 65 years, and 50% were female. Diabetes was the primary cause of 40% of ESRD cases, and hypertension was the primary cause of 30% of ESRD cases. Although adult blacks were 1.7 times more likely than whites to have diabetes and 1.4 times more likely than whites to have hypertension, blacks were 4.3 times more likely than whites to develop ESRD.

ESRD is a major public health burden, especially among blacks. Although a higher percentage of blacks than whites suffer from diabetes and hypertension, the racial disparity in the prevalence of ESRD is much greater. The incidence of ESRD might be reduced by 1) educating patients with diabetes and hypertension about the importance of diligent self-management and regular medical care, and by 2) encouraging physicians to monitor the renal function of their patients with diabetes and hypertension.

# Sub article: Developing, Implementing, and Disseminating Evidence-based Healthy Aging Programs in Community-based Organizations

**Published:** 2005-03-15

**Authors:** Nancy A Whitelaw, Nancy Erckenbrack, Robert Schreiber, June Simmons, Nancy Wilson

**Affiliations:** Center for Healthy Aging, The National Council on the Aging

## Track: Evidence-based Programs: Research, Translation, and Evaluation

Our objective was to convey findings on the development, implementation, and dissemination of evidence-based healthy aging programs in community-based organizations. Fifteen locations, including care management agencies, senior centers, and churches in Boston, Mass; Houston, Tex; Los Angeles, Calif; and Portland, Ore, implemented one of four evidence-based healthy aging programs. These sites reached a group of highly diverse participants, including Latinos, African Americans, Native Americans, Chinese, as well as other non-English–speaking populations.

The National Council on the Aging conducted a three-year national project to translate health promotion and disease management studies into evidence-based model programs that are feasible for local agencies to operate and attractive to older adults. Expert review panels examined the evidence for effective interventions on a variety of prevention topics and recommended the development of model programs for diabetes self-management, nutrition, depression management, and physical activity. Four regional teams across the country translated each of the review panel’s recommended evidence-based interventions into model programs, two using a lay-leader method and two designed for implementation by care managers. Throughout the translations process, experts from the Centers for Disease Control and Prevention and academic institutions reviewed the programs to ensure fidelity.

The results of this project were evaluated using the RE-AIM framework for assessment of health behavior interventions. Each site succeeded in reaching diverse populations with risk factors relevant to the model program. Approximately 350 older adults were reached during the pilot period. Data gathered on the pilots suggest that the programs were effective — improvements in self-reported health status were reported, and satisfaction levels were high. Of critical importance were the willingness and ability of various groups and participants to adopt the program. Considerable attention was paid to maintaining fidelity to the “proven” intervention during the implementation phases. On-site reviews by outside experts confirmed that implementation was consistent with original studies. Maintenance is still being assessed, but 12 of the 15 pilot sites continue to offer these programs.

The results of this project reinforce the belief that community-based organizations are highly capable of implementing evidence-based health promotion and disease management programs for older adults. Such programs can be incorporated into existing programming, providing excellent opportunities for organizations to expand their reach and quality of offerings. The four model programs are now offered as free toolkits, which guide organizations through the implementation of the programs.

# Sub article: Diabetes Awareness, Training, and Action Program: North Carolina’s Response to the Care of School Children With Diabetes Law

**Published:** 2005-03-15

**Authors:** Paula Hudson Collins

**Affiliations:** North Carolina Department of Public Instruction

## Track: Policy and Legal

In the fall of 2002, the Care of School Children with Diabetes Act was signed into law in the state of North Carolina. This law requires that all children with diabetes who are enrolled in any of the state’s public schools have an Individual Diabetes Care Plan available upon a parent’s request. The law also requires that schools enrolling children with diabetes provide general training on diabetes and its management for all faculty and support staff and two volunteer emergency care providers for students with diabetes.

To respond to the law, the North Carolina (NC) Department of Public Instruction, NC Department of Health and Human Services, NC Diabetes Advisory Council, American Diabetes Association, Wake Forest Baptist University Medical Center, Wake Area Health Education Centers, BlueCross BlueShield of NC Foundation, NC Healthy Schools, and the Diabetes Prevention and Control Branch of NC Public Health worked together to create the Diabetes Awareness, Training, and Action (DATA) Program. The DATA Program involved developing and producing training materials, designing and implementing six regional trainings across the state using a “train-the-trainer” model, seeking private funding for the project, developing the individual care plans and all reporting forms, informing parents, and preparing evaluation reports for various state agencies.

As a result of the DATA Program, all students with diabetes in North Carolina have a plan of care in place, which is determined by the primary care provider, school, and parent. Emergencies because of diabetes have decreased, and general awareness and acceptance of procedures related to diabetes control are better understood, accepted, and monitored in the school setting. The increased interaction among school personnel, medical personnel, and parents, especially parents of children in homes experiencing health disparities, have yielded the unintended benefits of improved communication and collaboration among these groups. In addition, this cooperative spirit has encouraged the community to work through doctors’ offices as well as public health clinics to make this diabetes information readily available to families that might not otherwise have the means to access health care.

An Individual Health Care Plan should be in place for children with any type of health care need. While the management of diabetes in children was the test case in North Carolina, children with any chronic condition should have access to care plans to avoid the need for a state law that relates to just one chronic condition.

# Sub article: Trying to Quit: Low-Income Smokers’ Access to Cessation Care in a Managed Care Environment

**Published:** 2005-03-15

**Authors:** Millicent Fleming-Moran, Kaigang Li, Joseph Gibson, Miriam Garland

**Affiliations:** Indiana University, Department of Applied Health Science

## Track: Methods and Surveillance

This study describes 295 smokers in a managed care safety-net insurance program, where 63% received cessation advice during at least one visit in the previous year. Our study asks: Does longer program enrollment increase a smoker's likelihood of receiving cessation advice?

The study population is drawn from Advantage program clients who are predominantly minority, working poor with Medicaid/Medicare or are under-insured county residents who meet 200% or less of federal poverty guidelines. State medical school practitioners coordinate the program in seven primary care clinics in a Midwestern urban county.

Telephone surveys using Bellview CATI survey software (Pulse Train Software, Ltd, Surrey, UK) were administered in English or Spanish to 731 Advantage enrollees. Of these, 317 were enrolled for less than one year, 281 were enrolled for one year, and 133 were enrolled for more than one year. Of the 731 enrollees, 295 (40.4%) were current smokers. The current smokers were categorized by sex, ethnicity, age, education, knowledge of primary care physician (PCP), and coronary heart disease (CHD) risk other than smoking. The association of each characteristic with cessation advice was determined by chi-square tests of significance. Predisposing factors (sex, age, ethnicity), enabling factors (education, known PCP), health care need (other CHD risk), and program enrollment time were tested in a logistic model of cessation advisement, using a forward selection process.

Advantage smokers who are female (72.0%), white (70.4%), and over age 65 (85.0%) and who know their PCP (68.5%) and have another CHD risk factor (89.3%) report more advice than smokers who are male (49.0%), minority (54.5%), and under age 35 (35.0%) and who do not know their PCP (51.0%) or have any other CHD risk factors (46.2%). Individuals who were enrolled for more than one year (71.2%) report more advice than individuals enrolled for less than one year (53.1%). In logistic analysis, other CHD risk doubled the likelihood of cessation advice (odds ratio [OR] 2.02; 95% confidence interval [CI], 1.5–2.8), as did being female (OR 1.95; CI, 1.1–3.3). Being over age 65 increased the likelihood of advisement (OR 1.5; CI, 1.09–2.12), while minority status reduced the likelihood (OR 0.41; CI, 0.24–.70). Enabling factors of education, enrollment time, or PCP recognition did not enter the model.

Safety-net programs increase access to and continuity of primary care in low-income communities where smoking is most prevalent. Advantage's 63% advisement rate exceeds that reported for other smokers using primary care and indicates appropriate outreach to high CHD risk smokers. More than one third (37%) of smokers in Advantage's program, however, report no cessation counseling. We propose examination of visit patterns, language difficulties, and clinical smoking records as ways to track and target younger, male, and minority smokers for provider prompts and cessation support. Increasing access to cessation care would reduce CHD, respiratory, and adverse reproductive outcomes in this population.
